# Research on Pathogenic Fungi and Mycotoxins in China (Volume II)

**DOI:** 10.3390/toxins16030114

**Published:** 2024-02-26

**Authors:** Tanvir Ahmad, Qi Zhang, Shihua Wang, Yang Liu

**Affiliations:** 1 School of Food Science and Engineering, Foshan University/National Technical Center (Foshan) for Quality Control of Famous and Special Agricultural Products (CAQS-GAP-KZZX043)/Guangdong Key Laboratory of Food Intelligent Manufacturing, Foshan 528231, China; tanvir@fosu.edu.cn; 2 Oil Crops Research Institute, Chinese Academy of Agricultural Sciences, Wuhan 430062, China; zhangqi01@caas.cn; 3 Key Laboratory of Pathogenic Fungi and Mycotoxins of Fujian Province, College of Life Sciences, Fujian Agriculture and Forestry University, Fuzhou 350002, China

The presence of pathogenic fungi and contamination of mycotoxins in food and feed pose significant threats and challenging issues to food in the world. Pathogenic fungal species, such as *Aspergillus*, *Penicillium*, *Fusarium*, and *Alternaria*, are major concerns in the food and feed industry due to their synthesis of mycotoxins, which are leading to significant economic losses in China [1,2,3]. Mycotoxins, which are naturally produced by certain fungi, can contaminate various crops as well as food and feed products under the specific conditions that occur during the phases of harvesting, post-harvest, and processing [4,5]. Mycotoxins are highly toxic substances and cause carcinogenicity, neurotoxicity, nephrotoxicity, immune toxicity, teratogenicity and hepatotoxicity in both humans and animals. These substances contribute to economic losses and create trade barriers for food products [6,7,8]. Among all the mycotoxins, the most toxic, prominent and concerning for both humans and livestock are Aflatoxins (AFs), Ochratoxin A (OTA), Deoxynivalenol (DON), Zearalenone (ZEN), Fumonisins (FB) and T-2 toxins [9,10,11]. These mycotoxins are subject to strict rules and regulations by national and international organizations worldwide to prioritize resources for mycotoxins risk assessment [12,13]. Recently, these mycotoxins have attracted a significant attention in China due to their frequent detection in various food and feed commodities [14]. Certain pathogenic fungi have the capability to produce multiple types of mycotoxins in one type of food or feed matrix. Consequently, the assessment of naturally occurring mycotoxin contamination in this matrix is very important to avoid contamination to ensure the safety and quality of these products [15]. Hence, this Special Issue, titled “Exploration of Pathogenic Fungi and Mycotoxins in China (Volume II)” (https://www.mdpi.com/journal/toxins/special_issues/Fungi_Mycotoxins_China_v2, accessed on 10 March 2023), was formulated to compile recent scientific progress on various pathogenic fungi and mycotoxins in China. In this Special Issue, a total of ten articles were published (nine research articles and one review article) by a diverse group of scientists from China. In this editorial, we present the scientific advances which highlighted in three portions based on the published articles (a) Developments in immunoassay detection methods for DON and understanding the Megabirnavirus influence on DON biosynthesis; (b) Regulatory mechanisms influencing growth, stress response, and Aflatoxin production in *Aspergillus flavus*; *and* (c) Mycotoxin biodegradation, OTA production mechanisms, and *Alternaria* toxins in food processing.

**Developments in Immunoassay Detection Methods for DON and Understanding the Megabirnavirus Influence on DON Biosynthesis**

*Fusarium* toxins represent a substantial group of mycotoxins, comprising over 140 known secondary metabolites produced by *Fusarium* species. DON is a highly toxic and prevalent mycotoxin found in a range of agro-food commodities. In this study, Mokhtar et al. (contribution 2) successfully generated a potent monoclonal antibody (mAb) by using hybridoma cell 8G2. The developed mAb exhibited a remarkable attraction to DON detection. Innovative on-site DON detection was achieved by using a 20 nm colloidal gold nanoparticle (AuNP) with a vLOD of 20 μg/mL, which was later surpassed by a 75 nm gold nanoflower (AuNF)-based strip with a more sensitive (vLOD of 6.67 μg/mL) presenting a threefold improvement over conventional AuNP-based strips. These advancements mark promising immunoassay methods for practical DON detection in agricultural products.

Mycoviruses can significantly reduce the host fungus virulence and production of its toxic secondary metabolites. In this context, Li et al. (contribution 1) identified a *Fusarium pseudograminearum* megabirnavirus 1 (FpgMBV1) capable of reducing the production of DON in *Fusarium pseudograminearum*. This was analyzed by HPLC-MS/MS. A comparative transcriptomic analysis revealed 2564 differentially expressed genes in the FpgMBV1-containing *F. pseudograminearum* strain in comparison to the virus-free strain. Among them, 1585 genes exhibited up-regulation while 979 genes displayed down-regulation. Specifically, the expression of 12 genes in a trichothecene biosynthetic gene (TRI) cluster was significantly down-regulated. This regulation presented specific metabolic process, offering insights into how the mycovirus FpgMBV1 decrease the DON production and TRI cluster gene expression. FpgMBV1 can be potentially introduced as a new detoxifying agent of DON.

**Regulatory Mechanisms Influencing Growth, Stress Response, Aflatoxin (Afs) Production in *A. flavus* and Management**

Rac (a member of the Rho family) plays a pivotal role in metabolism, apoptosis, angiogenesis, DNA damage responses, proliferation and immunosuppression across various species. In this study, Qin et al. (contribution 4) demonstrated that Rac deletion in *A. flavus* led to reduced mycilum growth, conidia production, pathogen virulence and AFB_1_ synthesis. The absence of Rac also prevented sclerotium formation in *A. flavus*. Rac-free strains exhibited heightened sensitivity to cell wall and osmotic pressure stress. Western blot analysis revealed the loss of phosphorylation in key proteins, indicating Rac’s role as a molecular switch in regulating Slt2-MAPK and Hog-MAPK cascade pathways. Overall, the role of Rac is very important for mycelium growth, conidia formation, AFB_1_ production, and stress response in *A. flavus*.

The primary function of glutamine synthetase (Gs) is to accelerate the conversion of ammonium and glutamate into glutamine. In addition, Gs is also involved in other biological functions. However, the roles of Gs in filamentous fungi are not fully understood. In this study, Wang et al. (contribution 6) found the conditional disruption of the glutamine synthetase (AflGsA) gene using a xylose promoter in *A. flavus* results in total glutamine deficiency. Glutamine supplementation restores the nutritional deficiency caused by AflGsA expression deficiency. AflGsA, regulated by the xylose promoter, influences spores and sclerotia development through the transcriptional control of genes. AflGsA maintains reactive oxygen species (ROS) balance and aids in oxidative stress resistance. It also regulates light signals by producing glutamine and exhibits glutamine synthetase activity requiring metal ions. In vitro, the inhibitor L-α-aminoadipic acid suppresses disrupting spores, sclerotia, and colony morphogenesis in *A. flavus*. These findings offer insights into the connection between nutrition metabolism, glutamine synthetase, and a potential strategy for fungal infection prevention.

*A. flavus* produces carcinogenic and mutagenic AFs, a risk of food safety and security by contaminating agricultural products. Hence, Wang et al. (contribution 8) characterized two bZIP (*AflatfA* and *AflatfB*) transcription factors and their genetic interaction. Deletion of *AflatfA* and *AflatfA/B* in *A. flavus* causes inhibition of mycelial growth but enhances the sclerotia production. The mutant strains, Δ*AflatfB* reduces sclerotia while Δ*AflatfA/B* increases conidia production. Mutants show delicate sensitivity to H_2_O_2_ and Δ*AflatfA* and Δ*AflatfA/B* strains reduce the conidia and AFB_1_ production on YES culture medium and host (corn and peanut) which revealed that *AflatfA* controls more cellular processes. The function of *AflatfA* is stronger than that of *AflatfB* during process regulation, except in the response to H_2_O_2_, where it might play a decisive role.

Histone 2-hydroxyisobutyrylation is a post-translational modification that can regulate secondary metabolism. In this context, Wang et al. (contribution 9) reported a novel histone 2-hydroxyisobutyryltransferase Afngg1 in *A. flavus* and explored its role in cell growth and AF biosynthesis. The findings of Wang et al. (contribution 9) suggested that Afngg1 is involved in histone 2-hydroxyisobutyrylation and chromatin modification. ΔAfngg1 mutant strain showed lysine 2-hydroxyisobutyrylation modification of histones H4K5 and H4K8 and inhibited mycelial growth. Remarkably, sclerotia production and AFB_1_ biosynthesis were entirely inhibited in the ΔAfngg1 mutant strain which may provide a new intention for AF contamination prevention.

An environmentally friendly, reliable and economical method for the inhibition of *A. flavus* and reduction of AF contamination is a challenging concern worldwide. A study by Yang et al. (contribution 10) reported that Ag-loaded titanium dioxide composites exhibited >90% *A. flavus* inhibition in light irradiation for 15 min. This method substantially reduce *A. flavus* contamination in peanut by preventing AF production. In this method, AFB_1_, AFB_2_ and AFG_2_ concentrations were decreased by 96.0%, 92.5% and 89.8%, respectively, without any adverse effects on peanut quality. This work provides useful information to construct an efficient and environmentally friendly method to inhibit the *A. flavus* and reduce the AF contamination. This findings are relevant/applicable to the field of food safety.

**Mycotoxin Biodegradation, OTA Production Mechanisms, and *Alternaria* Toxins in Food Processing**

Mycotoxins pose a significant threat to food safety and lead to diseases in humans and animals. Various detoxification methods reported, including physical, chemical, and biological. Among these, Ndiaye et al. (contribution 5) have reviewed recently developed biological methods involving microorganisms for mycotoxin detoxification by highlighting their reliability, cost-effectiveness, and implementation. However, it is difficult to understand the toxicity of the new metabolites resulting from mycotoxin biodegradation, as they can be either less or more toxic than the original compounds. Furthermore, Ndiaye et al. (contribution 5) also reviewed and explained the mechanisms used by microorganisms for mycotoxin control. This review article provides an overview of highly toxic mycotoxins, highlights the microorganism’s potential for mycotoxin detoxification, discusses screening methods for degradation compound, explores mechanisms of mycotoxin biodegradation, and offers practical applications in food and feed for mycotoxins biodegradation.

The biosynthesis of carcinogenic OTA by *Aspergillus niger* poses a significant health risk and also leads to potential economic losses in the food industry. In this study, Wei et al. (contribution 3) investigated *A. niger*’s production of OTA using different carbon sources. Six percent sucrose, glucose, and arabinose induced OTA biosynthesis, with 1586 differentially expressed genes evaluated to a non-inducing source. Genes related to OTA biosynthesis were up-regulated. AnGal4 identified in this study plays pivotal role for regulating the biosynthesis of OTA.

*Alternaria* is a filamentous fungus and responsible for food spoilage during storage and food processing. *Alternaria* species can also produce various types of mycotoxins. In this context, Qin et al. (contribution 7) reported the production of *Alternaria* toxins in processing tomatoes. *Alternaria* toxins (TEN, AME, TeA and AOH) and their conjugated toxins (AME-3-Glc, AME-3-S, AOH-3-S and AOH-9-Glc) were detected in *Alternaria*-infected processing tomatoes during storage. This study offers novel insights into potential presence of *Alternaria* toxins in tomatoes, providing valuable data for the prevention and control of these contaminants.
